# Non-Invasive Methods for Early Diagnosis of Endometriosis—A Comprehensive Narrative Literature Review

**DOI:** 10.3390/healthcare13243276

**Published:** 2025-12-13

**Authors:** Adriana Ioana Gaia-Oltean, Dan Boitor, Laura-Ancuta Pop, Geanina Galea, Teodora Telecan, Romeo Micu

**Affiliations:** 1 “Regina Maria” Hospital, 400117 Cluj-Napoca, Romania; oltean_adriana_ioana@yahoo.com (A.I.G.-O.); geaninagalea@yahoo.com (G.G.); romeo.micu@umfcluj.ro (R.M.); 2 Department of Obstetrics and Gynecology, “Iuliu Hatieganu” University of Medicine and Pharmacy, 400012 Cluj-Napoca, Romania; 3 Genomics Department, Medfuture—Institute for Biomedical Research, 400012 Cluj-Napoca, Romania; laura.ancuta.pop@gmail.com; 4 Faculty of Biology, University of Bucharest, 050095 Bucharest, Romania; 5 Department of Anatomy and Embryology, “Iuliu Hațieganu” University of Medicine and Pharmacy, 400012 Cluj-Napoca, Romania; t.telecan@gmail.com; 6 Department of Pathology, County Emergency Clinical Hospital, 400347 Cluj-Napoca, Romania

**Keywords:** endometriosis, early diagnosis, non-invasive testing, biomarkers

## Abstract

Endometriosis is a common gynecological pathology, with an incidence of nearly 10% in patients of reproductive age, and is still underdiagnosed. A thorough and well-spread diagnostic study of endometriosis based on epigenetic factor dysregulation can highlight potential areas for improvement. To quantify the potential and utility of non-invasive tools in the early diagnosis of endometriosis, an overview of current knowledge on epigenetic factors, based on DNA and RNA, is presented. Among these tools, it is important to highlight the role of miRNAs (microRNAs), cfDNA (cell-free DNA), and rRNAs (ribosomal RNAs), which are small molecules involved in endometriosis and numerous other pathologies. To evaluate their potential and utility in endometriosis, a salivary miRNA diagnostic test was conducted, the cfDNA methylation patterns of fragmented DNA circulating in bodily fluids (e.g., plasma) were analyzed, and cervical and uterine microbiomes were profiled for bacterial rRNA in patients with clinical suspicion of incipient endometriosis. Specific molecular profiles associated with endometriosis were analyzed. The first profile, a 109-miRNA saliva signature, was validated as a product of miRNA biomarkers and artificial intelligence modeling. In addition, peripheral blood cfDNA methylation biomarkers were identified by investigating nine genes in a molecular signature that requires validation. A profile was also obtained from cervical swabs and uterine washes, including molecular analysis of 16S rRNA amplicon sequencing to evaluate alterations in the cervical bacterial community. This review aims to optimize the integration of a non-invasive diagnostic tool for early endometriosis diagnosis. Genetic biomarkers can be correlated with clinical factors to improve diagnostic accuracy. Of the assessed diagnostic tools, salivary miRNA tests, a peripheral blood cfDNA methylation biomarker, and a microbiome rRNA signature may be useful for early diagnosis of endometriosis, as well as, implicitly, therapeutic attitude and follow-up.

## 1. Introduction

### 1.1. Endometriosis and Its Common Definitions

Endometriosis is a complex, chronic inflammatory disease characterized by the ectopic growth of endometrial-like tissue outside the uterus. With an estimated global prevalence of approximately 10% among reproductive-age women, it is recognized as a major public health concern that adversely affects quality of life and psychological well-being [1,2].

Endometriosis is mainly described as a condition that is estrogen-dependent and progesterone-resistant [3]. The etiopathogenesis of endometriosis and the development of epigenetic changes remain only partially elucidated. As a complex disorder, endometriosis is characterized by the interplay of multiple factors, such as retrograde menstruation, metaplastic transformation, endometrial progenitor cells, lymphatic and hematogenous spread, and mullerian remnant anomalies, as well as inflammation, oxidative stress, steroid hormones, and immunologic dysregulation. Previous genetic studies have largely been unsuccessful in identifying variants strongly associated with disease risk, but recent advancements in high-throughput technologies and molecular approaches have facilitated a more comprehensive characterization of the genetic risk factors implicated in endometriosis [4,5]. Clinically, in the context of heterogeneous pathology, endometriosis can present in different forms; its symptoms are integrated and correlated with three known phenotypes: ovarian endometrioma, superficial peritoneal endometriosis, and deep infiltrating endometriosis [6].

It is well established that genetic factors play a significant role in the pathogenesis of endometriosis. Imagistic techniques, specifically ultrasonography and magnetic resonance imaging, may facilitate the detection of endometriosis [7], which is challenging and often delayed. This is due to the difficulty of lesion detection, with the performance varying according to multiple factors, such as the radiologist’s experience, the complexity of the case, and access to technology [8,9]. Meanwhile, some lesions are too small or complex to be accurately evaluated. Nowadays, the treatment of endometriosis is complex, involving medical and surgical methods. Management depends on symptoms, age, stage, and, in specific cases, the need for preserving fertility [4,10].

### 1.2. The Current State of Endometriosis and Its Early Diagnosis

Despite its high prevalence and profound impact, endometriosis is still often undiagnosed or diagnosed when it has reached an advanced and hard-to-manage stage. Reliable non-invasive diagnostic methods for early diagnosis are lacking. On average, the prevalence of endometriosis in women of reproductive age is 10%, and in certain populations, it is up to 15% [2]; thus, early diagnosis of this pathology is necessary. To improve the diagnosis of endometriosis, an early, reliable, and non-invasive test is required.

Advances in genome-wide association studies (GWASs) and next-generation sequencing (NGS) have led to elucidation of the potential molecular mechanisms underlying disease pathogenesis and have facilitated the establishment of genetic risk prediction frameworks [11]. Advances in genomic sequencing technologies have further enabled the identification of biomarkers that can be used to monitor physiological and pathological states.

Recent advances in microbiome research have revealed compelling evidence linking microbiota dysbiosis to the pathogenesis and potential diagnosis of endometriosis. While endometriosis has traditionally been diagnosed using invasive procedures such as laparoscopy, emerging technologies that can evaluate alterations in the microbiota could serve as non-invasive tests for early detection and disease monitoring.

This review provides an overview of current knowledge on dysregulated epigenetic factors and dysbiotic changes in microbiota in endometriosis, highlighting potential areas that require further investigation and the implementation of validated findings.

## 2. Methodological Design

### 2.1. Study/Framework Aim

To integrate information regarding non-invasive methods for early diagnosis of endometriosis, a narrative review was performed. The aim of this review is to obtain an overview of current knowledge on dysregulated epigenetic factors and dysbiotic changes in the microbiota of endometriosis, highlighting potential areas for further investigation and the implementation of validated findings. For the narrative review, we propose integrating both qualitative and quantitative findings to synthesize existing knowledge and offer interpretive insights that connect evidence across contexts.

### 2.2. Search Strategy

A comprehensive literature search was conducted by accessing primary electronic academic databases: PubMed-MEDLINE—United States National Library of Medicine (NLM, 1996), Web of ScienceTM (WOS) (Clarivate Analytics, 1997), and Scopus (Elsevier, 2004). Dedicated and scientific terminology was applied, including “endometriosis”, “early diagnosis”, “non-invasive testing”, “biomarkers”, “epigenetic”, “gene”, “DNA methylation”, “histone”, “DNA mutations”, “RNA types”, “miRNAs”, “ribosomal RNA”, “gut microbiota”, “vaginal microbiota”, “reproductive tract”, “endometrium”, salivary test’’, “blood samples”, and “genital samples”. MeSH (Medical Subject Headings) was used to enhance precision, as well as Boolean operators “and”, “in”, and “or”. The main input targets a comprehensive coverage of relevant research articles. The main actions were identification, collection, and analysis.

### 2.3. Inclusion and Exclusion Criteria

The inclusion criteria are based on the current understanding of endometriosis, emphasizing early and non-invasive diagnosis. Parameters of interest include the genetic process and the involvement of the microbiota. Reviews and original articles were considered for analysis. All selected and analyzed articles were in English. The exclusion criteria included the following: articles for which the full text was not available, gray literature, and editorials or opinion pieces.

### 2.4. Selection of Evidence Source

All authors agree on the importance of an early diagnosis of endometriosis through means that are as non-invasive as possible, as well as the need to present an overview of what this means and why it is so important. For this narrative review, three reviewers conducted the literature search and article selection, following the inclusion criteria. The full text of articles matching the inclusion criteria was analyzed. Two of the reviewers carried out the review. Any disagreement between the reviewers was discussed and resolved through consensus.

### 2.5. Sustaining the Concept

In a narrative review design, the target priorities are represented through a synthesis of key themes, an interpretive discussion of what is known, and a conceptual exploration of future perspectives. International standards for narrative reviews were respected by emphasizing the importance of the data and explaining them, formalizing data extraction and reporting, ensuring clarity, and providing logical argumentation and interpretation with a relevant endpoint. All of these steps were inspired by the SANRA scale [12].

## 3. Fundamental Outcomes in Endometriosis

### 3.1. Genetics and Their General Implications

GWASs play a crucial role in identifying genetic variations linked to diseases. In this context, single-nucleotide polymorphisms seem to be significantly associated with endometriosis [13]. Genetic biomarkers may be DNA and RNA sequence variations associated with varying levels of disease susceptibility. Emerging data suggest that epigenetic crosstalk between DNA methylation and miRNA expression is widespread in various diseases. Notably, pre-miRNA transcription is epigenetically regulated, particularly through methylation of CpG islands in promoter regions [14].

Currently, evidence suggests that epigenetic interactions between DNA methylation and miRNA expression are prevalent in many pathologies. The transcription of pre-miRNA can be affected by epigenetic control, especially the methylation of the promoter-associated CpG island [14,15].

#### 3.1.1. Based on DNA

The expression of DNA epigenetics in endometriosis has been extensively studied. Endometriosis-related changes may occur and are responsible for DNA dysregulation. Changes in DNA methylation may contribute to the pathogenesis of endometriosis [2]. Genetic research is actively contributing to the development of a diagnostic test for genetic potential. The identification of DNA methylation biomarkers could help in understanding the cause of this pathology and facilitate its diagnosis. Of these, cell-free DNA (cfDNA) is starting to be considered as a promising biomarker for disease diagnosis, prognosis, and monitoring [16].

##### DNA Methylation Changes

DNA methylation is an epigenetic modification that plays a crucial role in regulating gene expression. This complex process involves the addition of a methyl group to a DNA molecule, typically at a cytosine base, and can alter gene expression without changing the underlying DNA sequence. Hypermethylation and hypomethylation are associated with upregulation and downregulation of gene expression [17], with genome-wide methylation profiling identifying 17,551 differentially methylated loci, among which 9777 were hypermethylated, and 7774 were hypomethylated [15].

In endometriosis, DNA methylation is one of the most studied and well-characterized pathogenetic processes [3,18]. Certain enzymes are responsible for DNA methylation and demethylation, namely DNA methyltransferases (DNMTs), which add a methyl group to cytosine. DNMT1 is the primary maintenance methyltransferase, and DNMT3A and DNMT3B catalyze de novo methylation [19]. Comparing cells from endometriotic lesions with eutopic endometrium from normal controls, methyltransferases DNMT1, DNMT3A, and DNMT3B were found to be upregulated in epithelial cells from endometriotic lesions compared to the eutopic normal ones [3].

cfDNA methylation analysis examines the methylation patterns of fragmented DNA circulating in bodily fluids, such as plasma. cfDNA methylation patterns can serve as biomarkers for various diseases, including endometriosis, and provide insights into the tissue of origin.

##### Histone Changes

Histones are proteins whose modifications affect how DNA is packaged and how genes are expressed, forming the nucleosome [3]. The nucleosome core contains a histone octamer with up to 2 units of histones H2A, H2B, H3 [20]. Histone proteins undergo various post-translational modifications, including methylation, acetylation, and ubiquitination, which can influence the transcription, repair, and replication processes [20]. Histone methyltransferases and demethylases, histone deacetylases, and histone acetyltransferases are enzymes that contribute to histone protein modifications [3,21]. Hypermethylation and hypomethylation are associated with the up- and downregulation of gene expression, and have been reported for several key genes associated with endometriosis, including *ESR2*, *PR-B*, *HOXA10*, aromatase [3], and the GATA family of transcription factors [22]. WNT signaling, PI3K-Akt, cell division-MAPK, cadherin signaling, angiogenesis, neurogenesis, immunity, gonadotropin-releasing hormone receptor route, and cancer development are also implicated in the pathology of endometriosis [23,24].

##### DNA Mutations

At the molecular level, endometriosis exhibits significant genetic diversity, potentially leading to genomic instability. Gene expression is a multifaceted mechanism by which the information encoded in genes is translated into proteins. Genetic markers correspond to DNA sequence variations that may indicate a higher predisposition to certain diseases. Several studies have reported genetic mutations that contribute significantly to endometriotic pathology. Regarding the genes involved, it is important to mention *ARID1A*, *KRAS*, *SIRT1*, *P53*, *BCL6*, *PTEN*, *PIK3CA*, and *CTNNB1* [3,18,25]. *ARID1A* is the most frequently mutated gene in many gynecological cancers, such as ovarian and endometrial cancers, as well as other types, such as gastric, bladder, and lung tumors [26]. Besides endometriosis, *ARID1A* is also essential for embryonic development. *KRAS* is a gene known for cancer-driver mutations reported in endometriosis [3,25]. At the same time, *KRAS* is a central regulator of histone deacetylase *SIRT1* and transcription factor *BCL6*. More specifically, *KRAS* induces an inflammation that activates *SIRT1*, a specific biomarker documented in all stages of endometriosis [27] that downregulates p53, which in turn regulates LIF expression. LIF proteins are essential for embryo implantation [3].

#### 3.1.2. Based on RNA

Transcription and translation are processes by which cells express the genetic information in their genes. Identical RNA copies can be synthesized from the same gene, and at the same time, each RNA molecule can indicate the synthesis of many identical protein molecules. At the same time, each gene can also be transcribed and translated into different proteins—in vast quantities for some and small quantities for others [28].

The cell produces several types of RNA. RNA molecules copied from genes that direct protein synthesis are called *messenger RNA (mRNA)*. Small nuclear RNA *(snRNA)* molecules contribute to the splicing of pre-mRNA to form mRNA. Ribosomal RNA *(rRNA)* molecules form the core of ribosomes, and transfer RNA *(tRNA)* molecules form the adaptors that select amino acids and hold them in place on a ribosome for incorporation into protein [28]. Non-coding RNAs *(ncRNAs)* do not contribute to protein translation. Among these, microRNAs (miRNAs) are small, single-stranded ncRNAs that are well studied in endometriosis and many other pathologies.

##### MicroRNA (miRNA)

miRNAs are small sequences of 19–22 nucleotides characterized by a non-coding capacity and with well-defined roles in post-transcriptional regulation of protein expression. They play an important role in the post-transcriptional regulation of messenger RNA (mRNA) and are derived from primary miRNA (pri-miRNA) transcripts through two sequential cleavage events [14].

The expression levels of miRNAs can be altered due to various factors. Changes in miRNA processing can be quantified as specific panels of upregulated and downregulated molecules [25]. Dysregulated miRNAs have been associated with a large number of human diseases, such as cancer, diabetes mellitus, cardiovascular disease, and neurodegenerative disease [14,25]. A specific panel of downregulated molecules constitutes a molecular signature. An increase in miRNA expression characterizes upregulation, whereas downregulation indicates a decrease in miRNA expression [21]. miRNAs have attracted considerable attention as potential epigenetic biomarkers in endometriosis. Research has identified distinct miRNAs whose expression is altered in endometriotic lesions relative to normal endometrium [8]. These dysregulated miRNAs have the potential to be detected in human fluids [16]. Most of them are in blood samples, offering the possibility of a minimally invasive approach [11], or in salivary samples, which are acquired via much more accepted and non-invasive methods [16].

##### Ribosomal RNA (rRNA)

Ribosomal RNA (rRNA) is the primary component of ribosomes, serving as the cellular machinery responsible for protein synthesis. It provides structural support for ribosomal proteins and is the most abundant type of RNA in the cell. It is a critical tool in understanding cellular function. In endometriosis, the most widely used method is 16S rRNA gene sequencing, which consists of total RNA extraction according to the manufacturer’s specifications, followed by amplification of the V4 region of the 16S rRNA gene and sequencing [9,29].

##### Long Non-Coding RNA (lncRNA)

LncRNA is a group of non-coding RNA transcripts longer than 200 nucleotides. Such non-coding transcripts are abundantly found in the human genome. Initially, data suggested that lncRNAs do not participate in protein coding; however, further research revealed that they can modulate gene expression through epigenetic, transcriptional, and post-transcriptional mechanisms. Increasing evidence indicates that lncRNAs play crucial roles in the initiation and progression of a wide range of diseases, particularly tumors [4]. Endometriosis is associated with differential expression of lncRNA [4,30]. LncRNA *MALAT1* is an lncRNA that has been widely studied, and it has been demonstrated that it participates in various physiological and pathological processes. In endometriosis, LncRNA *MALAT1* has shown upregulation in ectopic endometrial tissue, contributing to the modulation of endometriosis pathogenesis [4].

##### Gene Expression

Dysregulations in gene expression are quantified across a wide range of differences between eutopic endometrium and ectopic endometriosis lesions. Signaling pathways like PI3K-AKT, WNT, and MAPK [16,18] contribute to oxidative stress and focal adhesion, as well as HIF1α/NF κB, and YAP/TAZ/EGFR [16], which can occur as a cause or consequence of this disease. Some variants regulating the expression of genes involved in cell adhesion and proliferation, like *LINC00339*, *VEZT*, *FGD6*, *CDKN2BAS*, and *CDC42*, in blood and endometrium, increase the risk of endometriosis [18,31]. The expression of genes with critical roles in hormonal regulation (*ESR1*, *PGR*, *GREB1*) has also been implicated in endometriosis [18]. Of these, variants on chromosome 6 near ESR1 have been associated with endometriosis and various other reproductive traits and diseases. Chromosome 6 and its genetic variants in the region are highly correlated with the expression of *ESR1* and *PGR*, impacting the coregulation of these hormone receptor genes [32]. *GREB1* has also been associated with the transcriptional splicing of GREB1 in ovarian tissue. Splicing is a process in which introns are removed, and exons are joined together to form mature mRNA [33].

All these cell-type-specific genetic effects on gene expression and splicing underscore the need to investigate genetic effects in disease-relevant tissues to better understand and quantify how genetic risk factors regulate genes and increase endometriosis risk.

### 3.2. Microbiota and Its Possible Link to Endometriosis

#### 3.2.1. Gut Microbiota

Over the past few decades, the microbiome has been recognized as a critical determinant of human health. This gut microbiota includes over 100 trillion microbes—predominantly bacteria, but also archaea, viruses, fungi, and protozoa—with the majority residing in the colon. It plays essential roles in nutrient metabolism, vitamin synthesis, maintenance of intestinal barrier integrity, and, most notably, regulation of host immunity and systemic inflammation [34,35]. Disruption of this microbial balance, referred to as dysbiosis, has been implicated in the pathogenesis of numerous inflammatory conditions. Current evidence regarding microbial dysbiosis in endometriosis suggests that the presence of microbes within the peritoneal cavity may result from translocation via three primary pathways: the lymphatic system, the bloodstream, and the ascending route from the female genital tract [36,37].

Multiple studies have demonstrated significant shifts in the gut microbiota in women with endometriosis (Table 1). Among the prevailing theories of endometriosis pathogenesis, the “bacterial contamination hypothesis” posits that bacterial endotoxins, particularly lipopolysaccharides, play a significant role in promoting disease progression. Supporting this, studies have identified substantial contamination with *Escherichia coli* in both the menstrual blood and peritoneal fluid of women diagnosed with endometriosis [38]. A 2025 meta-analysis confirmed that women with endometriosis exhibit distinct gut microbiota dysbiosis, characterized by significantly lower alpha diversity compared to healthy controls, which is linked to elevated pro-inflammatory cytokine activity and impaired immune function [39].

Dysbiosis of the gut microbiota and altered microbial composition may contribute to the development of endometriosis. The depletion of *L. ruminococcus* in the gut might be a biomarker for endometriosis [9]. However, alterations in microbial composition have also been implicated in a variety of reproductive and systemic disorders, including polycystic ovary syndrome, infertility, inflammatory bowel disease, and neoplasms [40].

The mechanistic pathways linking gut dysbiosis to endometriosis are also influenced by the gut microbiota, which modulates several biological pathways relevant to endometriosis, including estrogen metabolism, immune modulation, and inflammatory cytokine production [6,41]. First of all, when we refer to estrogen metabolism, the gut microbial “estrobolome” regulates estrogen reactivation and systemic circulation. Dysbiosis can lead to elevated estrogen levels, promoting ectopic endometrial growth [37]. The second topic is highlighted by two articles published in 2014 and 2021. It refers to a disrupted gut microbiota that alters the Treg/Th17 balance and increases gut permeability. This allows translocation of bacterial endotoxins, such as lipopolysaccharide, into the bloodstream, which triggers systemic and peritoneal inflammation [42].

#### 3.2.2. Reproductive Tract Microbiota

Traditionally, the upper genital tract and peritoneal cavity were considered sterile environments. However, this notion has been increasingly challenged by recent studies examining the human microbiome across various anatomical sites [34,35] (Table 1). In healthy individuals, the genital tract microenvironment is tightly regulated, characterized by an anatomically intact mucosal barrier, a stable vaginal microbiota dominated by *Lactobacillus* species, a balanced hormonal milieu, and competent immune surveillance. The retrograde flow of menstrual blood may facilitate the translocation of microorganisms into the peritoneal cavity, potentially exacerbating inflammation and supporting ectopic lesion development. Flow cytometry analysis of ME revealed a significant reduction in uterine natural killer (NK) cells within both the CD45^+^ and CD45^−^ cell fractions in endometriosis patients compared with controls. RNA was collected and analyzed via PCR and RNA-Seq. Thus, these phenotypic characteristics of ME-derived cells may serve to gain deeper insight into its pathobiology and also as a non-invasive tool for diagnosing endometriosis [43].

The female reproductive tract represents a unique mucosal environment crucial for reproductive health. Historically, research has focused on the vaginal microbiota, dominated by *Lactobacillus* species that maintain a protective acidic environment. However, recent molecular studies demonstrate that the cervix and uterus also host resident microbial communities [35]. These discoveries challenge the long-standing paradigm of a sterile upper tract and suggest that microbiota throughout the reproductive tract play important roles in disease susceptibility, fertility, and pregnancy.

Although understudied, cervical microbiota seem to mirror vaginal profiles. The cervix acts as both a gateway and a barrier between the lower and upper reproductive tract. Its mucus plug and epithelial lining provide mechanical protection and immune defense. Studies on cervical microbiota indicate that its composition often resembles the vaginal environment but may be less strongly dominated by *Lactobacillus* species. Furthermore, cervical microbiota influences local immunity and cytokine production, thereby shaping susceptibility to infection and reproductive outcomes. This transitional niche underscores the significance of microbial balance at the interface between the vagina and the uterus. In infertile women undergoing IVF, dominance of *Lactobacillus* in cervical samples was correlated with positive pregnancy outcomes, while greater diversity and prevalence of pathogens like *Prevotella* and *Atopobium* decreases were also associated with endometriosis [9] and also with failed pregnancies [44].

The uterine cavity was traditionally considered sterile, based on the limitations of culture-dependent methods. However, sequencing approaches have detected bacterial DNA in endometrial tissue and fluid, suggesting the presence of low-biomass, but potentially functional, communities. Modification of the uterine microbial composition, including *Lactobacillus*, *Gardnerella*, *Atopobium*, *Prevotella*, and *Streptococcus* compositions, is a constant concern in patients with endometriosis [6]. These findings point to an active role for the uterine microbiota in regulating endometrial receptivity [45].

The vaginal microbiota is the most studied compartment of the reproductive tract. Vaginal dysbiosis means a disruption in this microbial equilibrium. It can compromise mucosal defenses, increasing susceptibility to lower genital tract infections and contributing to the development of severe gynecologic conditions, including cervical intraepithelial neoplasia and carcinoma [35,46]. In reproductive-age women, the vaginal environment is usually dominated by *Lactobacillus* species, particularly *L. crispatus*, *L. gasseri*, *L. jensenii*, and *L. iners*. These organisms produce lactic acid, hydrogen peroxide, and bacteriocins, which collectively maintain a low vaginal pH (<4.5) and inhibit colonization by pathogenic bacteria and viruses. When *Lactobacillus* dominance is lost, the microbial community becomes more diverse, with an increase in anaerobes such as *Gardnerella vaginalis*, *Atopobium vaginae*, *Prevotella* spp., and *Mobiluncus* spp. This state, known as bacterial vaginosis, in addition to endometriosis [6], is associated with increased susceptibility to sexually transmitted infections, pelvic inflammatory disease, and adverse pregnancy outcomes. Thus, the vaginal microbiota is not only a key barrier to pathogens but also a determinant of reproductive success [47].

In addition, the reproductive tract microbiota exhibits both intra- and inter-individual variability, shaped by physiological factors such as age, ethnicity, menstrual cycle phase, ovarian stimulation, and pregnancy. Lifestyle factors that influence the reproductive tract, including diet, sexual behaviors, and hygiene practices, exert a significant impact on the composition of the reproductive microbiota [46].

**Table 1:** Dysbiosis of reproductive tract and gut microbiota associated with endometriosis (different vs. common).

	Gut Microbiota	Reproductive Tract Microbiota (Vagina, Cervix, Endometrium, Endometriosis Lesions)
Different germs	*CLOSTRIDIALES CLOSTRIDIA* decreased [9] *CLOSTRIDIALE LACHNOSPIRACEAE* decreased [9] *LACHNOSPIRACEAE RUMINOCOCCUS* decreased [9,35] *FAECALEBACTERIUM* increased [9] *BIFIDOBACTERIUM* increased [6,9] *SHIGELLA* increased [9] *AKKERMANSIA* [9]	*ATROPOBIUM* absent (vagina) [34] *UREAPLASMA* increased (cervical, endometriosis lesions) [34,48] *PSEUDOMONAS* increased (endometriosis lesions) [48] *ALISHE WANELLA* increased (endometriosis lesions) [48] *SHIGELLA* increased (cervical) [34] *FIRMICUTES* increased [49] *ACTINOBACTERIACEAE* decrease (cervical) [49] *BACTEROIDES* decrease (cervical) [49] *DALIASTER*- decrease (deep endometriosis) [49]
Common germs	*LACTOBACILLUS* decreased (gut, vagina, cervix, endometrium, fallopian tube) [6,9] *ESCHERICHIA COLI* increased (gut, endometrium, cervical, vagina) [6,18,34,50] *STREPTOCOC* increased (gut, endometrium, cervix, endometrioma, deep endometriosis) [6,34,50] *ENTEROCOC* increased (gut, endometrium, endometriotic lesions) [6,50] *PREVOTELLA* increased (gut, endometrium) [9,51] *GARDNERELLA* increased (gut, cervical) [34]	

## 4. Clinical Implications of Biomarkers in Endometriosis

Endometriosis is a chronic inflammatory disease [48] that significantly contributes to pelvic pain [52] and infertility [53,54]. It is also a heterogeneous disease with different clinical presentations. The major challenge of this pathology is that adjunct diagnostic tools, including biomarker assessments and imaging techniques, often yield inconclusive results. Conceiving and applying functional genomic tools is difficult [54]. Moreover, note that all findings need to be validated in large and independent cohorts.

For this, NGS has transformed endometriosis diagnosis by offering more comprehensive and accurate genomic information [18,55]. Genomic studies based on NGS have enabled these biological parameters, known as biomarkers, to contribute to diagnostic and monitoring states.

Microbiome research has provided compelling evidence linking gut microbiota dysbiosis to the development and potential diagnosis of endometriosis. While endometriosis has traditionally been diagnosed through invasive procedures like laparoscopy, new findings indicate that specific changes in the gut microbiota could serve as non-invasive biomarkers for early detection and disease monitoring. Collectively, these findings support the hypothesis that gut microbiota composition not only reflects disease state but may also influence the clinical course of endometriosis. Emerging evidence underscores a complex bidirectional relationship between endometriosis and the microbiota.

These methods lack sufficient sensitivity for early-stage endometriosis and are limited in their applicability to advanced cases. Consequently, both therapeutic management and patient monitoring are compromised, resulting in frequent failures of conventional treatment strategies. This highlights the need for further investigation into biomarkers and microbiome-targeted therapies as part of a precision medicine approach to endometriosis.

### 4.1. Clinical Implications of DNA. Peripheral Blood cfDNA Methylation Profile

Genome-wide DNA methylation profiling includes multiple alterations in genes in both ectopic and eutopic endometrium, with direct implications for the pathogenesis of endometriosis [56,57]. It is essential to support new therapeutic approaches, but a definitive DNA test for endometriosis does not exist yet.

In a recent study, Benkhalifa et al. [16] quantified peripheral cfDNA and the specific differential methylation of a group of genes. The study features a sample of 78 young women, 38 of whom had endometriosis confirmed via laparoscopy and 40 of whom were healthy. A significant difference between the two groups was observed, with the results showing 3.9 times more cfDNA in the serum of women with endometriosis than in healthy women. In addition, nine target genes with different methylation profiles between the two groups were identified. These results were evaluated in terms of potential contributions and implications in the pathogenesis of endometriosis. The inconsistencies were also reported in studies assessing serum cfDNA levels, likely reflecting the challenges arising from conditions unrelated to endometriosis, including lifestyle influences and diverse biological variations. Consequently, cfDNA quantification alone is insufficient for diagnosing endometriosis. Incorporating epigenetic characterization of cfDNA enhances its potential utility as an early diagnostic marker [16].

### 4.2. Clinical Implications of RNA

Numerous studies have explored the diagnostic potential of circulating miRNA signatures within the context of endometriosis; however, the reported outcomes remain incongruent, largely attributable to methodological heterogeneity and inconsistencies in the selection of the control cohort [58,59]. In a similar vein, salivary miRNA has been extensively investigated by several research groups as a potential source of biomarkers for a wide range of benign and malignant pathologies, but its relevance within the context of endometriosis remains unexplored [60].

#### 4.2.1. miRNA Salivary Test

In endometriosis, the possible identification of a saliva micro-ribonucleic acid (miRNA) signature has unlocked new perspectives. A multicenter, prospective study confirmed the diagnostic utility of the saliva miRNA signature for this pathology. Moreover, the study sustained the reproducibility and stability of miRNA quantification and sequencing over time [8].

A prospective multicenter study involving 200 patients was initiated based on epidemiologic, clinical, and saliva-sequencing data. The target was a preliminary identification and validation of a saliva miRNA signature. Genome-wide miRNA expression profiling via small RNA sequencing using NGS was performed [8]. Salivary samples were obtained from women with dysmenorrhea or chronic pelvic pain aged between 18 and 43 years old, suggestive of endometriosis according to the protocol. Confirmations of endometriosis for the patients included laparoscopy, confirmed via histology; magnetic resonance imaging (MRI); or ultrasound showing deep endometriosis with colorectal involvement and/or endometriomas, confirmed via a multidisciplinary team applying established criteria. The 109-miRNA diagnostic saliva signature was thus validated using miRNA biomarkers and artificial intelligence modeling (AI). Of those 109 miRNAs, only 2 (miR-34c-5p and miR-19b-1-5p) have been reported before in endometriotic pathology. In addition, 29 of them (hsa-miR-511-3p, hsa-miR-34c-5p, hsa-miR-544a, hsa-miR-591, hsa-miR-4677-3p, hsa-miR-4328, hsa-miR-6133, hsa-miR-4510, hsa-miR-19b-1-5p, hsa-miR-1224-3p, hsa-miR-6875-3p, hsa-miR-6718-5p, hsa-miR-25-5p, hsa-miR-204-3p, hsa-miR-96-3p, hsa-miR-873-5p, hsa-miR-6839-5p, hsa-miR-629-5p, hsa-miR-520e, hsa-miR-518a-5p) have been mentioned as being associated with some of the major signaling pathways of endometriosis: PI3K/Akt, PTEN, and Wnt/β-catenin. Overall, among the 109 miRNAs in the molecular signature, 84 (77%) have been reported to be associated with different pathophysiologic pathways in benign and malignant disorders. The 109-miRNA saliva diagnostic signature had a sensitivity of 96.2% and specificity of 95.1% [8].

This study presents several limitations that warrant consideration. Foremost among them is the limited sample size of the interim analysis. Moreover, clinical factors such as disease phenotype, stage, endometriosis-related infertility, and adenomyosis were underrepresented, reducing statistical power, here referring also to complex cases, which require further standardization. The presence of concomitant pathologies, such as uterine myomas or inflammatory disorders, may have also contributed to potential bias in the findings. A potential source of bias that may arise from prior hormonal treatments, which could potentially influence miRNA expression, although the studies indicated that miRNA expression is not significantly altered either across the menstrual cycle or in response to sex steroid hormone therapies. This suggests that the diagnostic assay may be unaffected by contraceptive use and maintains the diagnostic test property as independent of hormonal treatment. Also, although 97.3% of the human miRNAs were detectable and subsequently analyzed in the salivary samples, it cannot be excluded that the remaining 2.7% of miRNAs may play a role in endometriosis. Lastly, the diagnostic signature demonstrated higher accuracy in patients over 18 years of age, where patients under this age were not represented in this study. Consequently, these findings cannot be directly extrapolated to younger populations. Such extrapolation may constitute an important factor to consider in efforts aimed at achieving earlier diagnosis of endometriosis. In current clinical practice, where the salivary miRNA test is already in use, several contraindications apply, as mentioned in ClinicalTrials.gov number NCT05244668. These include pregnancy, active infection at the time of sample collection, a medical history of HIV or cancer, and suboptimal sampling conditions. The latter may involve contamination with endogenous substances such as blood or nasopharyngeal secretions [8].

In a second step, a prospective, multicenter validation of the salivary miRNA signature was considered. The study targeted the diagnostic accuracy, reproducibility, and clinical utility of this miRNA signature for endometriosis. In this context, a population of 971 patients aged 18–43 years, with symptoms suggestive of endometriosis and an endometriosis prevalence of 77%, was included. The control diagnosis of endometriosis was managed via imaging, laparoscopy, or both. The saliva miRNA signature had an accuracy of 96.6% and sensitivity of 97.3% [61].

#### 4.2.2. Cervical and Uterine rRNA Microbiota Biomarkers

The dysbiosis of the microbiota is increasingly recognized as a potential non-invasive biomarker for diagnosing endometriosis. The importance and role of the microbiome are well debated, with implications in the formation and progression of endometriosis and in inflammatory pathways. This dysbiosis, which is described in endometriosis, is thought to be both causative and a consequence of the pathogenesis [15]. Profiles from cervical swabs and uterine washes have been used for molecular analysis of the V4 regions of 16S rRNA amplicon sequencing in women with endometriosis and compared to healthy controls [29,34,62]. Additional exclusion criteria for participants comprised age under 18 or older than 45 years at the time of sampling, pregnancy, postmenopausal status, antibiotic or probiotic use within the previous eight weeks, a diagnosis of inflammatory or functional bowel disorders, any history of gastrointestinal malignancy, and any abnormal Pap test result within the last three years [34]. This analysis revealed altered microbiota compositions and distributions along the female reproductive tract in women with endometriosis [29]. Notable changes include reduced microbial diversity and increased abundance of pro-inflammatory taxa such as Proteobacteria, Prevotella, and Erysipelatoclostridium, alongside decreases in beneficial genera such as *Lactobacillus*, *Ruminococcus*, and *Bifidobacterium* [34]. These microbial alterations may reflect underlying pathophysiological processes that are central to endometriosis, such as immune dysregulation, chronic inflammation, and estrogen metabolism dysfunction [29]. Importantly, fecal metabolite profiling has also identified compounds such as 4-hydroxyindole that are significantly reduced in endometriosis patients; this molecule has demonstrated anti-inflammatory effects and lesion-suppressive activity in preclinical models [63].

### 4.3. An Integrative Appraisal of Non-Invasive Tools in Endometriosis

The salivary miRNA has been examined by multiple research groups as a source of biomarkers for a variety of benign and malignant conditions; however, its application in the context of endometriosis has not yet been explored. This initial study, conducted on 200 participants, aimed to achieve an early diagnosis through a non-invasive miRNA-based approach that may enable more timely therapeutic intervention, thereby potentially mitigating disease progression and reducing the overall severity of endometriosis [8]. The test was validated and implemented, but still, a larger-scale test with more statistical power was required. Subsequently, a second prospective, multicenter study was undertaken to validate the salivary miRNA signature. This investigation enrolled 971 patients and aimed to assess the diagnostic accuracy, reproducibility, and clinical utility of the miRNA signature for endometriosis [61]. Still, the evaluation of the miRNA salivary signature for endometriosis as a diagnostic tool requires further large-scale studies. But, until then, the value of this test lies in evaluating a complex and heterogeneous pathology, such as endometriosis, where the combination of miRNAs’ intrinsic properties and AI’s modeling power can improve early diagnosis.

Additional promising strategies also included the assessment of the cfDNA methylation profile in peripheral blood samples. Additionally, genes implicated in endometriosis were identified, and their methylation profiles were subsequently evaluated. The study, including 78 young women, evidenced four times more cfDNA in the serum of women with endometriosis than in healthy women. Four genes were hypomethylated, indicating elevated expression levels independent of the stage of endometriosis [16]. This study is the first to report elevated circulating cell-free DNA from four genes with significant differential methylation in women with endometriosis, demonstrating reliability in detecting endometriosis. This opens new potential for preventive, early diagnosis of endometriosis, but needs validation and large-scale studies.

Emerging strategies for endometriosis diagnosis also involve rRNA in genital samples, in conjunction with microbiome profiling. In studies, cervical and vaginal samples were obtained after V4 regions of the 16S rRNA gene were amplified [29,34]. Differences at the gene level sustaining dysbiosis were observed, but further research is needed to determine the directionality and causality of this association, specifically whether dysbiosis contributes to the development of endometriosis or is a consequence of the disease. Collectively, these approaches aim to sustain the same target, namely the intention of being as non-invasive as possible, and sustaining an early diagnosis of the endometriosis. Of all this, the miRNA salivary test has a major benefit in that it is already validated and implemented. At the same time, it is the one that is the easiest to proceed, being as non-invasive as possible. An extension of the inclusion criteria for a younger age could represent a better sustaining of the principle of an early test.

**Figure 1:**
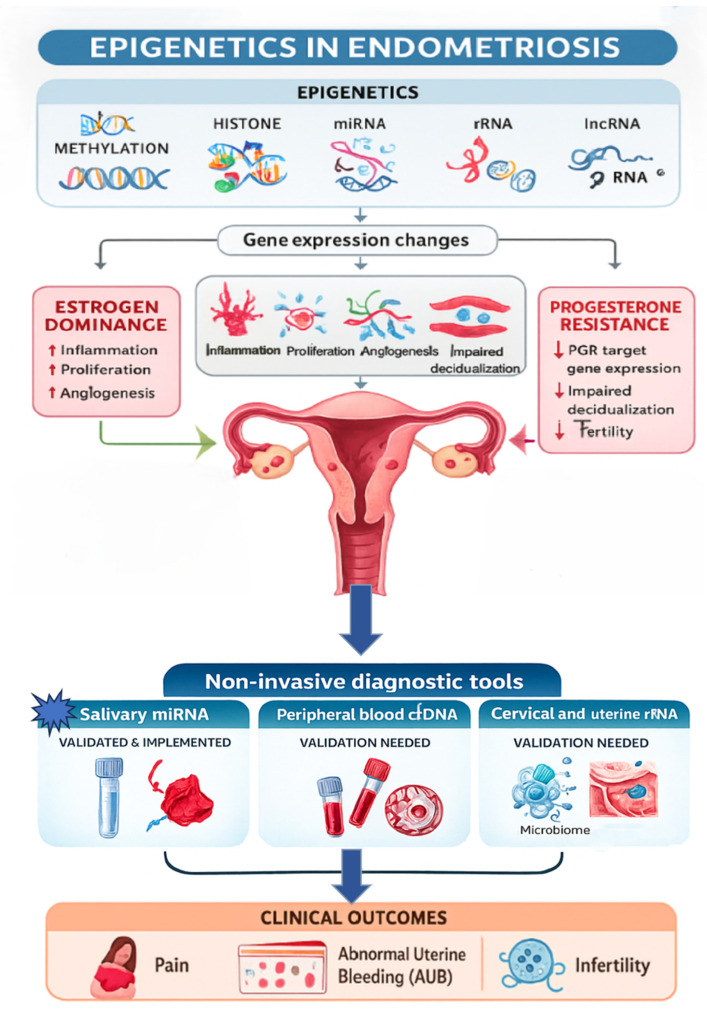
**Synthesis of non-invasive tools in endometriosis** [3,8,16,29,44,61]. Epigenetics plays a significant role in the development and progression of endometriosis through altering mechanisms like DNA methylation, histone modifications, and non-coding RNAs (miRNA, rRNA, lncRNA). These epigenetic changes influence key functions in ectopic endometrial-like cells, affecting processes such as proliferation and angiogenesis, modulating inflammation, and inducing estrogen dominance and progesterone resistance, leading to decreased fertility. However, because they are reversible, epigenetic mechanisms are promising targets for novel diagnostic biomarkers. Of those, the salivary miRNA test has been validated and implemented, proving more than useful in the early non-invasive diagnosis of endometriosis. The other two promising approaches are represented by the analysis of cfDNA from blood samples and rRNA from genital samples, in correlation with microbiome testing. All of these approaches aim to deliver an early and non-invasive diagnosis of endometriosis, limiting the clinical outcomes, as well as possible long-term clinical implications, as much as possible. This figure was produced with AUTOCAD, PAINT, and WORD.

A synthesis of non-invasive diagnostic tools in endometriosis is presented in Figure 1.

## 5. Conclusions

Epigenetics is increasingly important and is being considered in the quest to understand the causes and consequences of endometriosis. Thus, therapeutics targeting epigenetic mechanisms have been, and should continue to be, encouraged.

Methods such as miRNA expression, DNA methylation, and 16S rRNA amplicon analysis can influence gene expression patterns, potentially impacting endometriosis development. A tool for early and sensitive disease diagnosis could considerably improve the care of these women and enable preventive medicine. Developing an algorithm for stratifying patients at risk using a salivary miRNA test and rRNA bacterial community profiling may provide a useful diagnostic tool for the early diagnosis of endometriosis and for improving diagnostic strategies and, implicitly, therapeutic attitude and follow-up. The salivary miRNA test has been validated and partially integrated. DNA methylation and 16S rRNA amplicon analyses, although promising, have not yet been implemented.

This review aims to facilitate the integration of a non-invasive diagnostic tool for early endometriosis diagnosis. Genetic biomarkers can be correlated with clinical factors and imaging results to improve diagnostic accuracy. Those already validated as diagnostic tools require a more accepted implementation method, and validation in independent cohorts is recommended for those still being researched.

## 6. Future Directions and Trends

### 6.1. Improving Early Diagnostic Strategies for Novel Treatments

Nowadays, symptoms of endometriosis often begin at an early age, often followed by a 4–11-year delay in diagnosis, as consistently reported worldwide. Improving non-invasive diagnostic methods for endometriosis based on genetic findings can offer a more patient-friendly alternative to invasive procedures, thereby enhancing early detection and condition monitoring. Besides non-invasive RNA tests, validating non-invasive DNA tests and microbiota can improve the early detection of this pathology. Furthermore, these genetic findings may identify specific pathways or genes, serving as a stepping stone toward novel treatment strategies and more personalized care. The combination of two technologies (NGS and AI) provides a tool with reasonable accuracy for diagnosing endometriosis.

A non-invasive test based on miRNA has been validated [8,61]. This novel test could be applied for the early, non-invasive diagnosis of endometriosis, although large-scale validation is required. Future validation efforts may focus on a unique molecular signature based on the circulating free DNA levels and differential methylation profiling of nine hypomethylated genes. This strategy is promising for advancing predictive and preventive diagnostic tools for endometriosis. It also provides a good opportunity for evaluating 16S rRNA for a microbiome signature.

### 6.2. The Applicability of Non-Invasive Tests in Endometriosis Diagnosis in Adolescents—A Model of Preventive Medicine

For a young patient with severe symptoms and a lack of imagistic signs, a non-invasive genetic test can vastly change the evolution of this pathology, starting with an early diagnosis. Moreover, endometriosis often runs in families due to its genetic components, with first-degree relatives being at higher risk. Given that these factors more or less manifest as symptoms, their inclusion in a non-invasive test can shift our perspective on this disease, thereby improving care for these women.

## Data Availability

No new data were created or analyzed in this study.

## Abbreviations

The following abbreviations are used in this manuscript:

AI	Artificial Intelligence
AUB	Abnormal Uterine Bleeding
CfDNA	Cell-free DNA
DNMTs	DNA Methyltransferases;
GWAS	Genome-Wide Association Study
lnc RNA	Long non-coding RNA
cf DNA	Cell-free DNA
MRI	Magnetic Resonance Imaging
ME	Menstrual Effluent
mRNA	Messenger RNA
miRNA	MicroRNA
NGS	Next-Generation Sequencing
PGR	Progesterone Resistance
rRNA	Ribosomal RNA
snRNA	Small nuclear RNA
tRNA	Transfer RNA
